# Single Beat Determination of Intraventricular Systolic Dyssynchrony in Patients with Atrial Fibrillation and Systolic Dysfunction.

**DOI:** 10.5812/cardiovascmed.8776

**Published:** 2013-05-20

**Authors:** Anita Sadeghpour, Alireza Hoghooghi, Zahra Alizadehsani, Mohsen Rezaei, Sevil Aghapour, Majid Haghjoo

**Affiliations:** 1 Echocardiography Research Center, Rajaie Cardiovascular Medical and Research Center, Tehran University of Medical Sciences, Tehran, IR Iran; 2 Cardiac Electrophysiology Research Center, Rajaie Cardiovascular Medical and Research Center, Tehran University Medical Science, Tehran, IR Iran; 3 Rajaie Cardiovascular Medical and Research Center, Tehran University of Medical Sciences, Tehran, IR Iran

**Keywords:** Atrial Fibrillation, Heart Failure, Systole, Auditory neuropathy

## Abstract

**Background:**

Atrial fibrillation (AF) is the most common clinically significant cardiac arrhythmia. However, diagnosis of intraventricular dyssynchrony in patients with AF is difficult due to beat-to-beat variation. Additionally, evaluation of mechanical dyssynchrony in the traditional method is based on average of 5 to 10 beats, which is exhausting and time consuming. Single-beat evaluation of a beat with equal subsequent cardiac cycles has been proposed as an accurate method in patients with AF.

**Objectives:**

We proposed to evaluate intraventricular mechanical dyssynchrony by measuring time-to-peak systolic velocity between basolateral and basoseptal segments (septum to lateral wall delay) using Tissue Doppler Study (TDI) by two different methods.

**Materials and Methods:**

31 patient (68 ± 10.3 years) with heart failure (EF < 35%) and AF rhythm, R-R cycle length more than 500 msec were evaluated. We found a target beat in which preceding R-R (R-R1) to pre-preceding R-R (R-R2) ratio was 1(RR1/RR2 = 1) then measured the intraventricular dyssynchrony in that cycle. Intraventricular dyssynchrony was also determined and averaged for 8 consecutive cardiac cycles. The values at RR1/RR2 = 1 were compared with the average of intraventricular dyssynchrony in eight cycles and the relationship between dyssynchrony were evaluated by paired T-test, linear Pearson correlation (r2), linear regression analysis.

**Results:**

The average of dyssynchrony in eight cycles showed a positive correlation with dyssynchrony in target beat RR1/RR2 = 1. Average of dyssynchrony in target beat was 46.77 msec, and average of 8 cycle was = 47.701, (P value = 0.776, Pearson linear correlation 0.769).

**Conclusions:**

Measurement of intraventricular dyssynchromy in basoseptal and basolateral segments in AF and heart failure patients in a single beat with RR1/RR2 = 1 , were very similar to the average value of eight cardiac cycle.

## 1. Background

Cardiac resynchronization therapy (CRT) has emerged as an established therapy for patients with systolic heart failure (HF), low ejection fraction (EF) and prolonged QRS duration and who had received optimal drug treatment (1, 2). CRT improves left ventricular function, clinical status, quality of life and reduces hospitalization and mortality (3). Atrial fibrillation (AF) is a common arrhythmia in HF patients with increasing morbidity and mortality (4, 5). Although the CRT trial patients were chiefly diagnosed mostly included patients with sinus rhythm, there are a number of studies that have reported HF patients with AF will improve with CRT treatment (6, 7). Despite the tendency to use this therapy, there is non-response in about one-third of the patients (8). It is proposed that electrical dyssynchrony as determined by electrocardiography may not be an accurate indicator of electromechanical delay; a number of studies have compared electrical with mechanical dyssynchrony criteria and established that the mechanical dyssynchrony has better predictor response to CRT rather than electrical dyssynchrony (9, 10). It is also revealed that tissue Doppler imaging parameter, the peak systolic velocity (PSV) of the septum-to-lateral wall difference may be better selected responders for CRT(11). Evaluation of mechanical dyssynchrony in the traditional method is based on averaging a random number of consecutive cardiac cycles that are exhaustive and time consuming. On the other hand, there has been an interest in using a single-beat with equal subsequent cardiac cycles to assess left ventricular systolic function. Therefore, we expect that single-beat determination can be used as a practical and quick method to estimate dyssynchrony parameters during AF.

## 2. Objectives

The purpose of this study was to evaluate and determine the use of single single-beat with equal subsequent cycle and to assess LV dyssynchrony in patients with HF and AF.

## 3. Materials and Methods

### 3.1. Patient Selection 

Upon approval by the Ethics Committee in our research center, 31 patients with heart failure and atrial fibrillation were prospectively enrolled in this 6-month study. The inclusion criteria were congestive heart failure, class III-IV of New York Heart Association (NYHA) LV systolic dysfunction (EF ≤ 35%) and R-R cycle lengths longer than 500 milliseconds. We excluded patients with other causes of non-sinus rhythm, prosthetic valve disease and complete atrioventricular block. In addition, patients who had poor acoustic windows were excluded. Written informed/release consents was obtained from all patients. All patients underwent a standard 12-lead electrocardiography and echocardiographic examination for specific evaluation of intraventricular dyssynchrony. Heart rates were maintained between 60 and 100 beats/ min, during echocardiography.

### 3.2. Electrocardiographic Analysis

The standard method with 12-lead electrocardiograms was used, at a paper speed of 25 mm/s and a scale of 10 mm/mV. QRS period duration were measured (recorded from the surface leads demonstrating the greatest values). Two electrophysiologists assessed the QRS morphology and their diagnosis was considered final. They were blinded to the clinical status of the patients.

### 3.3. Echocardiographic Protocol

The patients were imaged in the left lateral decubitus position using a commercially available system (Vivid 7; General Electric Company, Norway) equipped with a 2.5-MHz phased array transducer. Left ventricular end-systolic, end-diastolic volumes and LV ejection fractions were assessed by biplane Simpson's method and graded according to established ASE/EAE guidelines. The images were obtained with a sweep speed of 100 cm/s, with gains and filters optimized. TDI was performed from 2-chamber, 4-chamber, and apical long axis views with an optimal Doppler insonation angle to assess myocardial regional function. TDI measurements were obtained from two different methods. At first, 3, 5 and 8 consecutive beats were stored digitally. The digital cine loops were analyzed using off-line analysis. We calculated mean of 3, 5 and 8 beats based on first beat. In the second method, we found a target beat in which preceding R-R (R-R1) to pre-preceding R-R (R-R2) ratio was 1 (RR1/RR2 = 1). Beats with R-R interval difference less than 5% of the mean have been considered as RR1/RR2 = 1 (12), then we measured intraventricular dyssynchrony in that cycle. The values at RR1/RR2 = 1 were compared with average of intraventricular dyssynchrony in 3, 5 and 8 cycles. All the echocardiographic examinations were performed by a board-certificated echocardiologist.

### 3.4. Determination of Intarventricular Dyssynchrony

**Figure 1.**
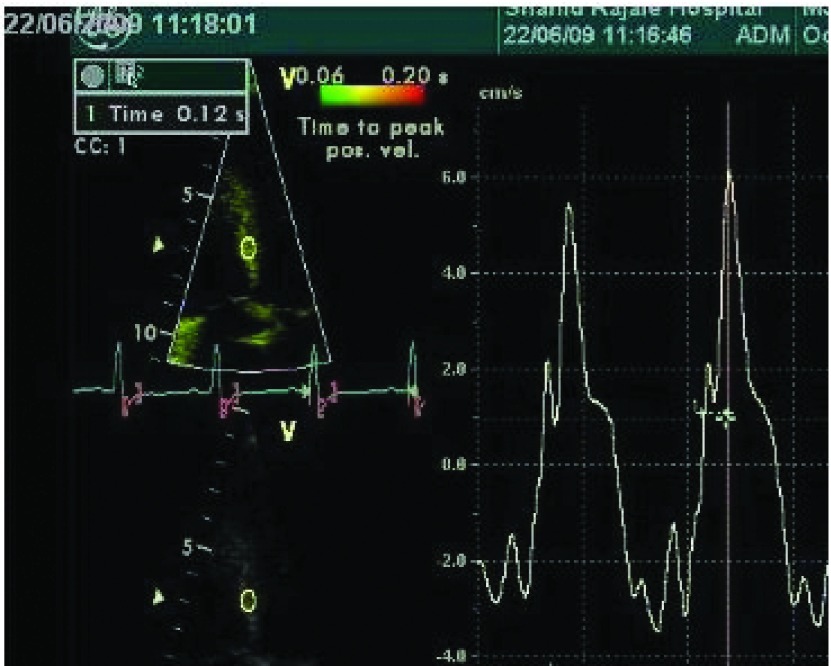
Intraventricular Dyssynchrony Assessment by Measuring Time from Q Wave to Peak Systolic Velocity of the Basal Septum

The interval from the beginning of Q-wave to the maximum positive velocity during the ejection period was defined as time to peak systolic velocity (Figure 1). Intraventricular dyssynchrony was assessed by placing the sample volume in the basal portions of the septum and lateral wall; the time to peak systolic velocity was measured in the septum and lateral wall, and the septal-to-lateral delay in peak velocity was considered as an indicator of LV dyssynchrony. A delay > 65 ms between the peak systolic velocity (PSV) of the septum-to-lateral wall was defined as a cutoff value for LV dyssynchrony (13). 

### 3.5. Statistical Analysis

SPSS 15 for Windows (SPSS Inc., Chicago, IL, USA) was applied for all analysis. Characteristic variables were expressed by mean ± standard deviation (SD), frequency, and relative frequency for scale and categorical variable respectively. Agreement between the calculated value of LV systolic dyssynchrony at R-R1/R-R2 and the measured average value of dyssynchrony in 3,5 and 8 consecutive cardiac cycles evaluated by using Bland and Altman plot in the linear regression line. P values of < 0.05 were considered statistically significant.

## 4. Results

### 4.1. Characteristic Data

Thirty-one patients (13 (42%) female, mean ± SD of age 68.35 ± 10.3 years) were enrolled in this study. Mean ± SD of LVEF was 22.74 ± 6.738. Mean ± SD of LVES was 137.48 ± 47.724 and mean ± SD of LVED was 173.42 ± 51.905. The most common cardiac diagnosis was coronary artery disease in 23patients (74.2%), 3 patients (9.7%) had dilated cardiomyopathy, 2 patients (6.5%) had hypertrophic cardiomyopathy and 3 patients (9.7%) had valvular heart disease.

### 4.2. Dyssynchrony Indices

**Figure 2.**
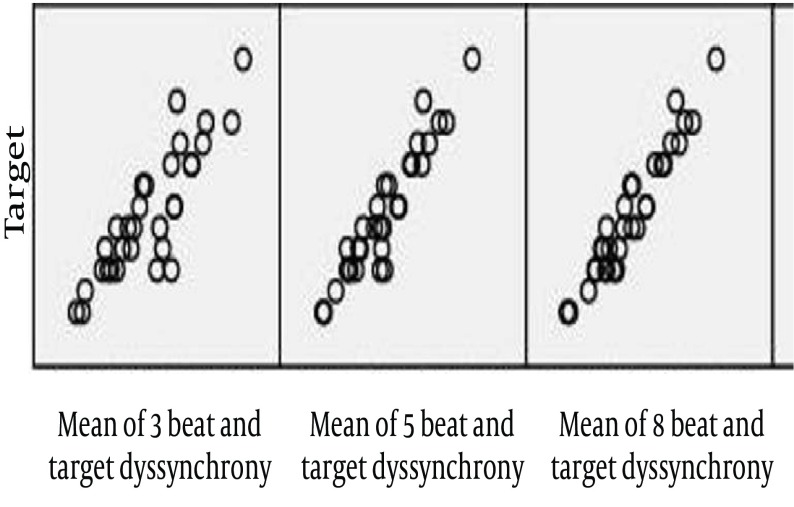
Increasing Positive CorrelationBetween the Measured Average Value of 3, 5 and 8 Consecutive Cardiac Cycles And the Calculated Value at R-R1 in the Linear Regression Line for SLWD

**Figure 3.**
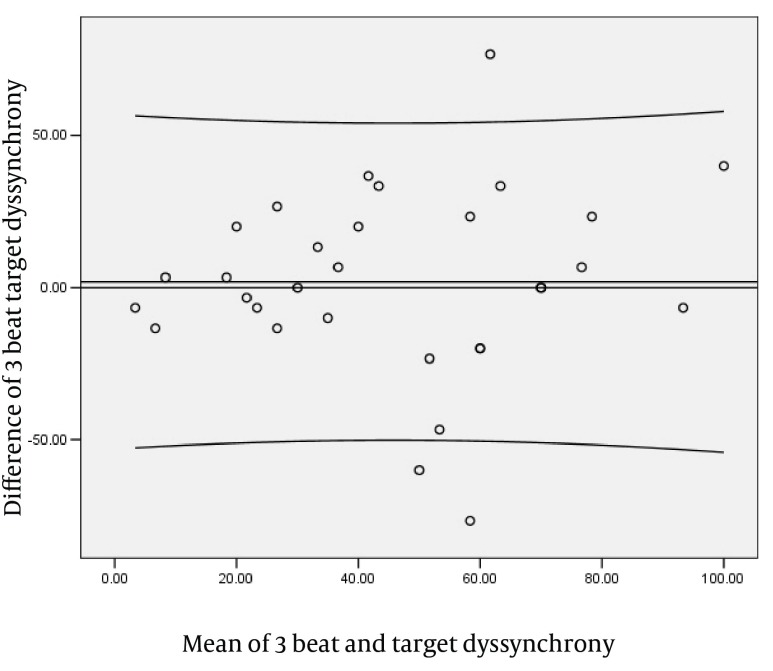
Blend Altman Diagram for3 Beats and Target Dyssynchrony

**Figure 4.**
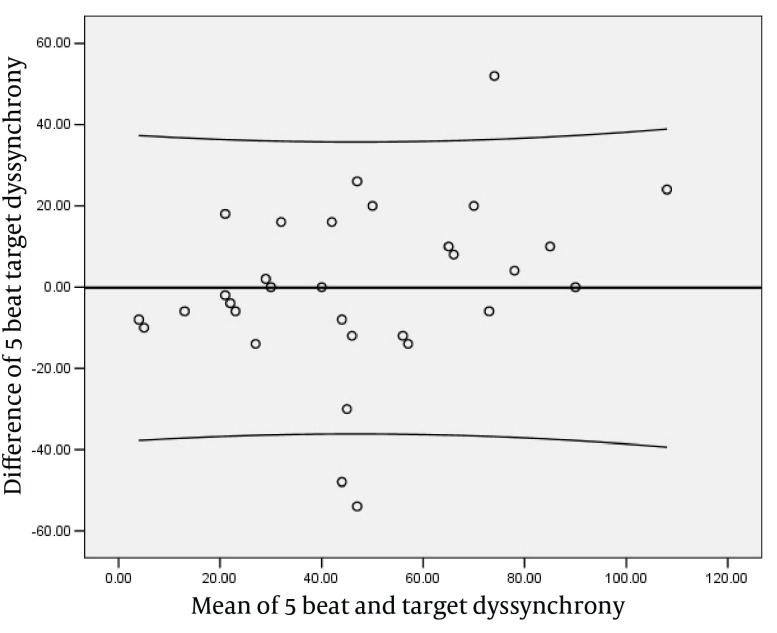
Blend Altman Diagram for5 Beats and Target Dyssynchrony

**Figure 5.**
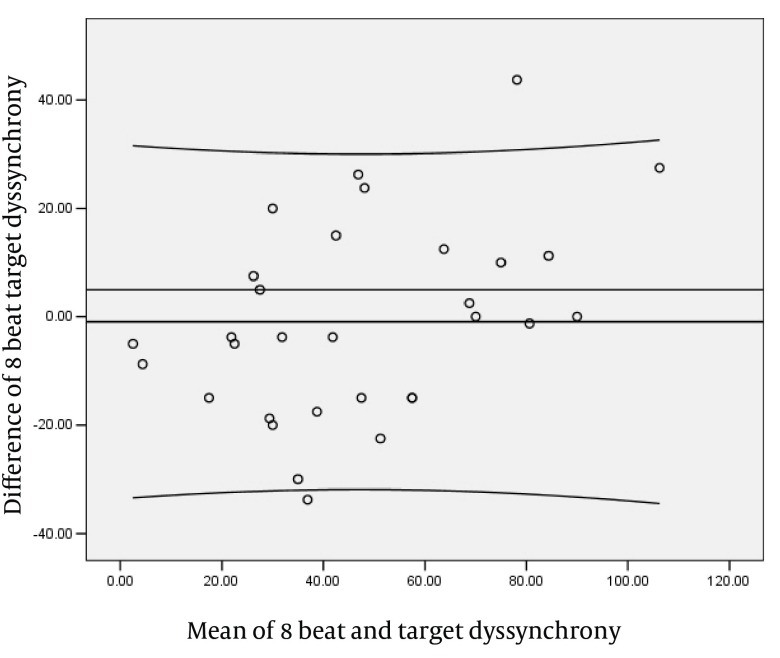
Blend Altman Diagram for 8 Beats and TargetDyssynchrony

The parameters of left ventricle systolic dyssynchrony (SLWD) during AF showed a significant positive correlation between the R-R1 interval and the averaged value of 3, 5 and 8 consecutive cardiac cycles. There were no significant difference between the target beat and the averaged value of 3, 5 and 8 consecutive cycles. There was an increasing trend in correlation coefficient between the target beat and the averaged value of 3, 5 and 8 consecutive cycles with the increasing in the number of beats (Figure 2). The regression equation for each of them is shown in Table 1. The Bland and Altman analysis showed a good relationship and agreement between the calculated and averaged values of dyssynchrony LV systolic parameter. (Figure 3-5). 

**Table 1.** The Mean, Correlation Coefficient and Regression Equation for 3, 5 and 8 BeatDyssynchrony with Target Beat

	Dyssynchrony		Correlation		Regression Coefficient		
	**Values ^a^**	**P value ^b^**	**Coefficient ^c^**	**P value **	**Intercept**	**Beta Coefficient**	**P value**
Target	46.77 ± 30.26 (0-120.00)	-	-1	-	-	-	
Average 3 beats	44.83 ± 27.71 (6.67-96.67)	0.720	0.459	< 0.009	α = 24.296	β = 0.501	< 0.001
Average 5 beats	47.032 ± 24.43 (8.00-96.00)	0.945	0.729	< 0.001	α = 4.298	β = 0.903	< 0.001
Average 8 beats	47.701 ± 23.13 (5.00-92.50)	0.776	0.806	< 0.001	α = -3.540	β = 1.055	< 0.001

^a^Values are Mean ± SD (min-max)

^b^Paired t test compared with target beat

^c^Correlation with target beat

## 5. Discussion

This study demonstrated that in AF rhythm, intraventricular dyssynchrony in a single beat with RR1/RR2 = 1 is quite similar to the average value of 8 cardiac cycles. Intraventricular mechanical dyssynchrony is characterized by abnormality in the movement of different LV walls due to delayed electrical conduction (14). It has become clear that delayed events during the cardiac cycle have a significant role in LV dysfunction. Since the identifying and quantifying of these events are the main prognosticator of response to CRT, various techniques are presently being studied to detect and quantify LV dyssynchrony. During AF, irregular pattern with the ventricular cycle length (R-R-intervals changes all mechanical and hemodynamic parameters from cycle to cycle (15, 16). Therefore obtaining the accurate values of LV systolic parameters via the standard protocol involves averaging a random number of consecutive cardiac cycles.

The mean number of cardiac cycles required in AF is more than that in sinus rhythm, this is both difficult and time consuming. Dubrey et al. showed that the mean number of cycles necessary to assess cardiac output in AF was three times that needed in sinus rhythm (17). Furthermore, the averaged value is still inaccurate due to the differences between the various windows this can reduce measurement certainties. Cardiac contractility is not constant during AF because of the force-interval relationships that determine mechanical restitution and post-extra systolic potentiation. It means that a shorter interval leads to a weaker beat and a longer interval leads to a stronger beat (16, 18-20). In addition, it has been reported that the ratio of RR1/RR2 intervals during AF accurately estimates the average value over all cardiac cycles (21-26). All measures of ventricular contractile function are affected by beat-to-beat variation (21). Beat-to-beat contractility during AF can be estimated from the ratio of preceding and prep receding R-R intervals (RR1/RR2) (23, 27, 28). In general, our results are consistent with those of other similar studies in as much as they demonstrate the value of R-R1/R-R2 = 1 as a good substitution for averaging values. Various echocardiographic techniques such as TDI have been used to assess intraventricular dyssynchrony (29). An observational multi-center study (Predictors of Response to CRT), reported the limited role of velocity dyssynchrony measurements by color DTI in predicting response to CRT (29). That study, however, had several important limitations such as enrollment of patients who did not meet criteria for CRT (20% of patients with EF > 35%), overall low feasibility and reproducibility of measurements, and usage of ultrasound systems and software from different vendors, including systems that had lower temporal resolution than the time intervals to be measured (30). In this study, we demonstrated that LV systolic dyssynchrony in patients with heart failure and AF could be accurately assessed using the ratio of the preceding and pre-preceding R-R intervals. We found that measurement of intraventricular dyssynchrony in basoseptal and basolateral segments in a single beat with RR1/RR2 = 1 by tissue Doppler echocardiography was similar to the average value of 8 consecutive cardiac cycles. In addition, there was a relation and agreement between the measured average value of 8 consecutive cardiac cycles and the calculated value at R-R1/R-R2 in the linear regression line for SLWD. In AF rhythm, despite beat to beat variation, measurement of intraventricular dyssynchromy by assessing a single beat with preceding R-R (R-R1) to pre-preceding R-R (R-R2) ratio 1 (RR1/RR2 = 1) is quite similar to the average value of 8 cardiac cycle.
